# Hypothermia modulates myeloid cell polarization in neonatal hypoxic–ischemic brain injury

**DOI:** 10.1186/s12974-021-02314-9

**Published:** 2021-11-13

**Authors:** Marina Seitz, Christian Köster, Mark Dzietko, Hemmen Sabir, Meray Serdar, Ursula Felderhoff-Müser, Ivo Bendix, Josephine Herz

**Affiliations:** 1 Department of Pediatrics I, Neonatology & Experimental Perinatal Neurosciences, University Hospital Essen, University Duisburg-Essen, Hufelandstr. 55, 45147 Essen, Germany; 2 Center for Translational Neuro-and Behavioral Sciences (C-TNBS), University Hospital Essen, University Duisburg-Essen, Essen, Germany; 3 Department of Neonatology and Pediatric Intensive Care, Children’s Hospital, University of Bonn, Bonn, Germany; 4 German Centre for Neurodegenerative Diseases, Bonn, Germany

**Keywords:** Microglia, Macrophages, Neonatal hypoxia–ischemia, Hypothermia, Myeloid cell polarization, M1 M2 polarization

## Abstract

**Background:**

Neonatal encephalopathy due to hypoxia–ischemia (HI) is a leading cause of death and disability in term newborns. Therapeutic hypothermia (HT) is the only recommended therapy. However, 30% still suffer from neurological deficits. Inflammation is a major hallmark of HI pathophysiology with myeloid cells being key players, participating either in progression or in resolution of injury-induced inflammation. In the present study, we investigated the impact of HT on the temporal and spatial dynamics of microglia/macrophage polarization after neonatal HI in newborn mice.

**Methods:**

Nine-day-old C57BL/6 mice were exposed to HI through occlusion of the right common carotid artery followed by 1 h hypoxia. Immediately after HI, animals were cooled for 4 h or kept at physiological body core temperature. Analyses were performed at 1, 3 and 7 days post HI. Brain injury, neuronal cell loss, apoptosis and microglia activation were assessed by immunohistochemistry. A broad set of typical genes associated with classical (M1) and alternative (M2) myeloid cell activation was analyzed by real time PCR in ex vivo isolated CD11b^+^ microglia/macrophages. Purity and composition of isolated cells was determined by flow cytometry.

**Results:**

Immediate HT significantly reduced HI-induced brain injury and neuronal loss 7 days post HI, whereas only mild non-significant protection from HI-induced apoptosis and neuronal loss were observed 1 and 3 days after HI. Microglia activation, i.e., Iba-1 immunoreactivity peaked 3 days after HI and was not modulated by HT. However, ex vivo isolated CD11b^+^ cells revealed a strong upregulation of the majority of M1 but also M2 marker genes at day 1, which was significantly reduced by HT and rapidly declined at day 3. HI induced a significant increase in the frequency of peripheral macrophages in sorted CD11b^+^ cells at day 1, which deteriorated until day 7 and was significantly decreased by HT.

**Conclusion:**

Our data demonstrate that HT-induced neuroprotection is preceded by acute suppression of HI-induced upregulation of inflammatory genes in myeloid cells and decreased infiltration of peripheral macrophages, both representing potential important effector mechanisms of HT.

**Supplementary Information:**

The online version contains supplementary material available at 10.1186/s12974-021-02314-9.

## Introduction

Neonatal hypoxic–ischemic (HI) brain injury is a leading cause of childhood mortality and neurodevelopmental morbidity [1]. Affecting 1–6 per 1000 newborns in Western societies with a mortality of up to 30%, it represents an enormous individual and familial burden with high socioeconomic costs. Frequent consequences are cerebral palsy, epilepsy, auditory and visual impairment as well as cognitive and motor deficits in later life [1, 2]. To date, therapeutic hypothermia (HT) is the standard therapy (ILCOR guidelines 2015). However, 30% of cooled infants still suffer from major neurological problems [3], because of a short therapeutic window (first 6 h of life), limited efficacy in more severe cases and a high number needed to treat [4]. This is supported by our previous experimental findings that protection of secondary injury processes and long-term cognitive deficits is limited by acute HT [5]. In spite of tremendous efforts to identify new and/or additional treatment strategies, translation into clinical practice is still challenging. Experimental and clinical studies showed that anti-apoptotic and anti-excitatory agents, such as insulin-like growth factor-1, the noble gas xenon and dizocilpine failed to provide greater protection than HT alone [6–8]. In support of this, we have previously shown in a murine model of HI-induced brain injury that potential interactions with regenerative stem cell therapies may occur due to alteration of the brain tissue environment following HT [9]. These studies demonstrate that a better knowledge of hypothermia’s effector mechanisms is needed to guide the rational design of combination treatments.

Inflammation is a major hallmark of HI pathophysiology, with myeloid cells playing a key role, participating in progression or resolution of injury-induced inflammation. Functional differences in myeloid cells are traditionally ascribed to coexisting diverse phenotypes with classically activated pro-inflammatory M1 cells and alternatively activated anti-inflammatory M2 cells. While M1 cells are supposed to contribute to neurodegeneration, M2 cells are suggested to promote repair and regeneration [10]. In addition to these cell types further immunoregulatory phenotypes have been described [11]. We and others have experimentally shown that HI induces pronounced differences in the expression of typical M1 and M2 markers in the injured brain [12, 13]. However, analyses were mainly performed in total tissue lysates, which is limited, since typical M1 and M2-related molecules, such as pro- and anti-inflammatory cytokines, are not exclusively expressed by myeloid cells. Furthermore, despite of the elegant possibility to perform selective analysis on ex vivo sorted CD11b^+^ myeloid cells from injured brains in different perinatal brain injury models, including traumatic brain injury, peripheral inflammation, HI and perinatal stroke [14–17], the composition of sorted cells with regard to their origin, i.e., peripheral monocytes/macrophages vs. resident microglia, remained largely unknown.

Even though anti-inflammatory effects of HT and inhibition of microglia activation have been proven in different models of HI-induced brain injury [9, 18], its impact on myeloid cell polarization is unknown. In an adult ischemic brain injury model, it was previously shown that pharmacologically induced hypothermia reduces mRNA expression of typical M1 markers, such as IL-23, IL-12 and iNOS, while M2 marker expression, e.g., Fizz-1, Ym-1 and Arg-1 were increased [19]. In one of our recent studies, we also observed a significant reduction of the pro-inflammatory M1 markers IL-6 and TNF-alpha following HT after neonatal HI [9]. Nevertheless, in both studies, total brain lysates were analyzed at a single timepoint, impeding clear cut conclusions about the time-dependent modulation of myeloid cells after HT.

In the present study, we investigated the effect of HT on the temporal and spatial dynamics of microglia/macrophage cell polarization 1, 3 and 7 days after neonatal HI via immunohistochemistry and gene expression analyses in ex vivo isolated CD11b^+^ myeloid cells. Flow cytometry analyses were used to get further insight, to which extent peripheral myeloid cells and resident microglia contributed to alterations observed in the total CD11b^+^ cell population isolated from HI-injured brains.

## Methods

### Animal care and group allocation

Experiments were performed in accordance with the ARRIVE guidelines with government approval by the State Agency for Nature, Environment and Consumer Protection North Rhine-Westphalia. C57BL/6 J mice, initially ordered from Charles River were bred in house and kept under a 12 h light/dark cycle with food and water ad libitum. Bodyweight was recorded at postnatal day 9 (P9), P10, P11 and P16. A total of 157 animals (81 female, 76 male) derived from 19 litters were enrolled. Thirty-three mice (16 female, 17 male) underwent sham surgery. Out of the remaining 124 exposed to hypoxia–ischemia 7 animals (5.7%, 2 female, 5 male) died during hypoxia. No mortality was observed afterwards until sacrifice of animals 1, 3 and 7 days after HI. For all analyses, animals per litter and experiment were randomly assigned to 3 experimental groups (sham, HI + normothermia (NT), HI + HT) prior to intervention. For immunohistochemistry, pups of one litter were further randomly divided to two timepoints of analyses prior to experimentation. Due to small litter size of mice and required pooling of samples for cell sorting, animals of one litter with 3 experimental groups (sham, NT, HT) could not be further divided into different timepoints of analysis. To control the potential influence of weight and sex, a stratified randomization was performed followed by simple randomization within each block to assign pups to individual groups. Individuals involved in data analysis knew the animals' designation but were blinded to group assignment. The first set of mice (31 female, 26 male) was used for histology and immunohistochemistry analysis. The second set of animals (47 female, 43 male) was used for magnetic activated cell sorting, followed by flow cytometry and mRNA expression analyses. Additional 3 (1 female, 2 male) mice were used for immune cell isolation and immediate flow cytometry analysis.

### Neonatal hypoxia–ischemia and therapeutic hypothermia

Hypoxic–ischemic brain injury was induced in 9-day-old animals as previously described [5, 9, 20, 21]. Briefly, the right common carotid artery was occluded through cauterization (high temperature cauter, 1200 °C, Bovie, USA) under isoflurane anesthesia (1.5–2.5 Vol%) followed by 1 h hypoxia (10% O_2_) in an air-tight oxygen chamber (OxyCycler, Biospherix, USA) after 1 h recovery with their dams. According to our previous study, where we identified a physiological body core (i.e., nesting) temperature of 35 °C for P9 mice [5] animals were placed on a warming mat (Harvard Apparatus, USA) to maintain nesting temperature during hypoxia. Sham-operated animals received anesthesia and neck incision only. Perioperative analgesia was ensured by subcutaneous administration of 0.1 mg/kg buprenorphine. Based on our previous reports [5, 9], HT was applied immediately following HI for 4 h with a target temperature of *T*_rectal_ 32 °C. Mice were placed on a custom-made plate with temperature control by water circulation. Controls (NT) were placed on a warming mat to maintain physiological body core temperature (*T*_rectal_ 35 °C) conforming to our previous work [5, 9].

### Tissue processing, histology and immunohistochemistry

1, 3 or 7 days after HI, mice were deeply anesthetized by intraperitoneal (i.p.) injections of chloralhydrate and transcardially perfused with ice-cold PBS followed by perfusion with 4% PFA. Brains were removed, post-fixed in 4% PFA overnight followed by dehydration in 30% Sucrose for 24 h. Brains were snap frozen in isopentane on dry ice. Tissue injury was assessed and scored on cresyl violet stained 20 µm cryostat sections as previously described [5, 9, 20, 22] with minor modifications. Briefly, 9 regions were scored: the anterior, middle and posterior cortex, CA1, CA2, CA3 and dentate gyrus of the hippocampus, the striatum and the thalamus. Each region was given a rating from 0 to 3 (0—no detectable cell loss or 0% infarct area (1 day after HI), 1—small focal areas of neuronal cell loss, ventricle enlargement at the striatal level or 10–40% infarct area (1 day after HI), 2—columnar damage in the cortex or moderate to severe cell loss in the other regions or 40–60% infarct area (1 day after HI), 3—cystic infarction and gliosis or 60–100% infarct area (1 day after HI)). The sum score from different regions was calculated for each animal resulting in a total maximum score of 27. Tissue atrophy was determined by measurement of tissue areas in ipsi- and contra-lateral hemispheres at a distance of 400 μm using Image J software (NIH, USA). Tissue loss was determined by comparison with contralateral volumes according to the following equation: 1−(volume ratio (left vs. right)) × 100.

For evaluation of cellular degeneration, neuronal loss, microglia activation and polarization, 20 µm cryostat sections taken at the hippocampal level (−1.9 to -2.0 mm from bregma) were stained according to our previous reports [5, 9, 21]. Briefly, tissue sections were thawed at 37 °C for 15 min and incubated with 1% BSA, 0.3% cold fish skin gelatin (Sigma Aldrich, Germany) in 1% Tween 20 in Tris-buffered saline (TBS-T) for 1 h at room temperature followed by primary antibody incubation at 4 °C over night. The following primary antibodies were used: rat anti-mouse NeuN (1:100, Millipore), rabbit anti-mouse/rat Iba-1 (1:1000, Wako), rat anti-mouse CD86 (1:200, abcam), goat anti-mouse CD206 (1:100, R&D Systems). Antibody binding was visualized by incubation with appropriate Alexa Flour 488 or Alexa Flour 555 conjugated secondary antibodies (anti-rat/goat/rabbit, all 1:500, all Thermo Scientific) for 2 h at room temperature. Nuclei were counterstained with 4′,6-Diamidin-2-phenylindol (DAPI, 100 ng/ml; Molecular Probes, USA). Cellular degeneration was determined by staining of DNA fragmentation using terminal transferase dUTP nick end labeling (TUNEL) according to the manufactures’ protocol (In situ Cell Death Detection Kit, Roche, Switzerland).

### Quantification of cellular degeneration, neuronal loss, microglia activation and polarization

Cellular degeneration and neuronal loss were quantified in TUNEL and NeuN-stained tissue sections via fluorescence microscopy according to our previous protocols [5, 9, 21]. Nine defined non-overlapping regions of interest (ROI, each 61.220 µm^2^, 3 ROI in the cortex, 3 ROI in the hippocampus and 3 ROI in the thalamus) were visualized by fluorescence microscopy (40 × objective; Axioplan; Zeiss, Germany) connected to a CCD camera (Axiocam ICc1, Zeiss). Mean values for each region and animal were calculated. Since neuronal density is not modulated in the contralateral hemisphere in our model [5], values of the ipsilateral hemisphere were related to contralateral values. Single cell counting was not possible in the contralateral hemisphere due to dense neuronal cell density in the neuronal layer. Therefore, NeuN^+^ areas were quantified and % neuronal loss was calculated according to the following equation: 100-((NeuN area ipsilateral*100)/NeuN area contralateral)). For quantification of TUNEL^+^ cells, unbiased software based object detection (Image J, NIH, USA) was used to determine the number of degenerating cells in the ipsilateral hemisphere. Confocal imaging (A1plus, Eclipse Ti, with NIS Elements AR software, Nikon, Germany) was used for assessment of microglia activation and polarization in Iba-1 and CD86/CD206 stained tissue sections. Using the 20 × objective z-stack images of 10 µm thickness (1 µm focal plane distance) were acquired in 7 ROIs (each: 99.729 µm^2^, 3 ROI in the hippocampus, 2 ROI in the thalamus, 2 ROI in the cortex). Images were converted into maximal intensity projections for quantification. CD86 single, CD206 single and CD86/CD206 double positive cells were counted. Single object counting was not possible for Iba-1 staining due to intensive local accumulation of microglia in severely injured regions and animals. Therefore, positively stained areas were quantified as a measure of cell density and activation.

### Processing of brain tissues for flow cytometry and magnetic activated cell sorting (MACS)

1, 3 and 7 days after HI, animals were deeply anesthetized by i.p. injections of an overdose chloralhydrate followed by transcardial perfusion with ice-cold PBS and removal of brains. Two hemispheres were pooled per sample to get a sufficient amount of CD11b^+^ cells after MACS. Brain tissues were mechanically and enzymatically dissociated using the Neural tissue dissociation kit followed by myelin removal according to the manufacturer's instructions (Miltenyi Biotech, Germany). The cell suspension was incubated with anti-CD11b coupled magnetic microbeads followed by magnetic separation of CD11b^+^ cells. A small aliquot of sorted cells (1–2 × 10^5^) was used for flow cytometry to determine purity, cellular composition and polarization. The remaining cells were centrifuged, and cell pellets were frozen at −80 °C until further processing. For comparison of the impact of different tissue homogenization and cell isolation procedures on the composition of isolated cells, brain hemispheres were dissected and homogenized through a 70 µm cell strainer (BD Biosciences) by continuous rinsing with 15 mL of cold HEPES-buffered RPMI1640. Brain samples were centrifuged at 400*xg* for 10 min at 18 °C. The supernatants were discarded and the pellets were resuspended in 15 mL of 37% Percoll in 0.01 N HCl/PBS and centrifuged at 2800*xg* for 20 min. Myelin was removed and the remaining cell pellet was washed twice in PBS followed by flow cytometry.

### Flow cytometry

Isolated cells were incubated with anti-CD45 Alexa Fluor700 (1:200, clone: 30-F11, BD Biosciences), anti-CD11b PE-Cy7 (1:800, clone M1/70, eBioscience), anti-Ly6G FITC (1:100, clone 1A8, BD Biosciences), anti-CD86 PE (1:400, clone GL-1, BD Biosciences), anti-CD206 Brilliant Violett (1:50, clone C068C2, Biolegend) for 30 min at 4 °C. Dead cells were excluded by staining with 1 µg/ml propidiumiodide (PI) prior to measurement. Brain resident brain microglia were identified as CD11b^+^CD45^intermediate^ and infiltrated peripheral myeloid leukocytes as CD11b^+^CD45^high^, which were further differentiated into neutrophils (Ly6G^+^) and macrophages (Ly6G^−^). Gates were set according to isolated cells prior to CD11b sorting. Myeloid cell polarization was evaluated by quantification of CD86 and CD206 single and double positive cells in the identified macrophage and microglia population. Gates were set according to fluorescence minus one (FMO) controls.

### mRNA expression analysis

For mRNA expression analyses in sorted CD11b^+^ cells, total RNA was isolated with the RNeasy Micro Kit (Qiagen Germany) according to the manufactures recommendations. First strand complementary DNA was synthesized using 0.6 µg of total RNA and TaqMan reverse transcription reagents (Applied Biosystems/Thermo Fisher Scientific). Polymerase chain reaction (PCR) was performed in duplicates in 96 well optical reaction plates for 40 cycles with each cycle at 94 °C for 15 s and 60 °C for 1 min using the StepOnePlus Real Time PCR system (Applied Biosystems/Thermo Fisher Scientific). PCR products were quantified using assay on demand primers and fluorogenic reporter oligonucleotide probes (Applied Biosystems/Thermo Fisher Scientific, Additional file 1: Table S1). CT values were normalized to the housekeeping gene beta-2-microglobulin [ΔCT = CT (target gene)-CT (beta-2-microglobulin)] and related to the mean of sham-operated animals using the ΔΔCT formula [ΔΔCT = ΔCT (sham) -ΔCT (HI+NT/HT)]. Fold change values were calculated.

### Statistical analysis

Results are expressed as box plots with individual data points including median values, the 25% and the 75% percentile or as bar graphs with mean ± standard deviation. For statistical analysis, the GraphPad Prism 6.0 software package (GraphPad Software) was used. Data were analyzed by Two-way ANOVA with one factor timepoint (d1, d3, d7) or brain region (cortex, hippocampus, thalamus) and the other factor treatment (sham, HI + NT, HI + HT). Whenever significant main or interaction effects were observed, post hoc analysis with Tukey’s multiple comparison test regardless of means of factor 1 and factor 2 was applied. In all analyses, *p* < 0.05 was considered statistically significant. Correlation analyses between the number of apoptotic cells/neuronal loss and Iba-1 immunoreactivity was performed with Pearsons’ or Spearmann’ correlation analyses depending on Gaussian distribution. For graphical presentation, individual data points and regression curves are shown.

## Results

### Neuroprotective effects of hypothermia develop over time

**Fig. 1:**
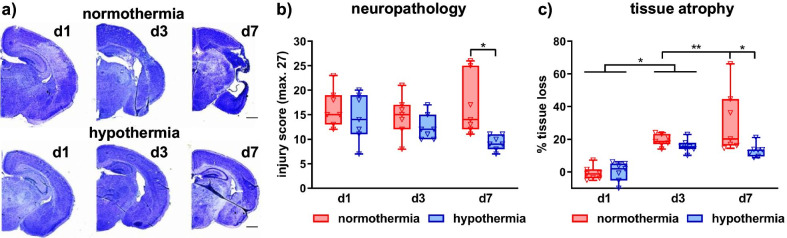
Neuroprotection by hypothermia develops over time. Histological brain injury was determined in cresyl violet stained tissue sections 1, 3 and 7 days after HI, performed in 9-day-old C57Bl/6 mice. Animals were exposed to 4 h hypothermia immediately after HI. Controls were kept at physiological body core temperature (normothermia). Representative images of injured hemispheres for each experimental group are shown in **a**, scale bar: 1 mm. Injury scores were assessed in different brain regions (cortex, hippocampus, thalamus and striatum) resulting in a sum score, which was quantified for each animal (**b**). Brain atrophy was analyzed by measurement of intact tissue volumes at a distance of 400 μm between + 1 mm and − 2.6 mm from bregma and volumes were calculated for total hemispheres (**c**). Tissue loss is expressed as the percentage of volume reduction compared to intact contralateral volumes (**c**). *p < 0.05, **p < 0.01, *n* = 7/treatment and timepoint

To assess the impact of immediate HT on HI-induced brain injury, brain tissue sections were analyzed for neuropathological alterations and tissue atrophy in cresyl violet stained tissue sections (Fig. 1a). In spite of a slightly reduced neuropathology in HT-treated animals 3 days after HI, most pronounced and significant protective effects were observed 7 days after injury, resulting from a gradual decrease in HT-treated animals between 1 and 7 days following HI (Fig. 1b). Furthermore, while NT animals revealed a gradual increase in HI-induced tissue loss between 1 and 7 days after HI, tissue loss was constant from day 3 onwards in HT-treated animals resulting a significantly reduced brain atrophy 7 days after HI (Fig. 1c).

**Fig. 2:**
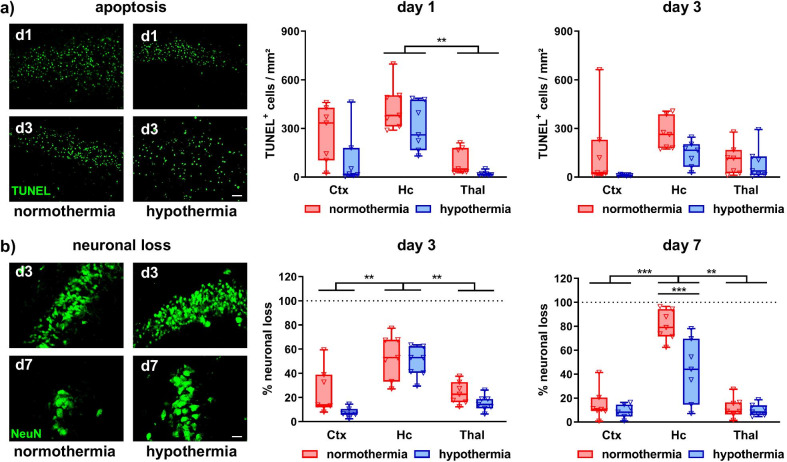
Hypothermia protects from delayed neuronal cell death. Nine-day-old C57BL/6 mice were exposed to HI followed by 4 h hypothermia or normothermia. Animals were sacrificed and analyzed via immunohistochemistry 1, 3 or 7 days after HI. Cellular degeneration (**a**) and neuronal loss (**b**) were assessed by quanfication of TUNEL^+^ cells and NeuN^+^ areas, respectively, in the cortex (Ctx), hippocampus (Hc) and thalamus (Thal). Representative images in **a** are derived from the CA1 (NeuN) and CA2 (TUNEL) regions of the hippocampus, scale bar: 20 µm. **p < 0.01, ***p < 0.001. *n* = 7/treatment and timepoint

To verify these results at the cellular level, we determined the number of degenerating cells and neuronal loss in different brain regions via immunohistochemistry for TUNEL and NeuN, respectively (Fig. 2). The most pronounced cellular degeneration was observed in the hippocampus, independent of treatment (Fig. 2a). Besides slightly reduced numbers of degenerating cells in the cortex and hippocampus in HT-treated animals at 1 and 3 days after HI, no significant differences were detected between NT and HT groups (Fig. 2a). In line with analyses of cellular degeneration, the hippocampus revealed the highest degree of neuronal loss 3 and 7 days after HI (Fig. 2b). In spite of a slight decrease by HT treatment in the cortex and thalamus 3 days after HI, no significant differences were observed at this timepoint (Fig. 2b). However, we detected a significantly reduced neuronal loss in the hippocampus of HT-treated animals 7 days after HI, which resulted from a gradual increase in neuronal loss between 3 and 7 days in NT control animals (Fig. 2b).

### Hypothermia alters expression of typical M1 and M2 marker proteins in a time and region-specific manner

**Fig. 3:**
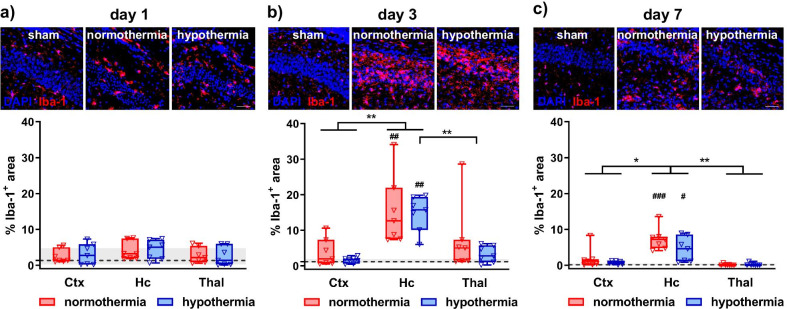
Microglia activation peaks 3 days after HI, but is not modulated by HT. Microglia activation was analyzed via immunohistochemistry for Iba-1 on day 1 (**a**), 3 (**b**) and 7 (**c**) after HI, performed in 9-day-old C57BL/6 mice. Animals were exposed to 4 h hypothermia immediately after HI. Controls were kept at physiological body core temperature (normothermia). Iba-1 immunoreactivity was quantified by measurement of positively stained areas in the cortex (Ctx), hippocampus (Hc) and thalamus (Thal). Representative images were acquired in the CA2 region of the hippocampus, scale bar: 50 µm. Dashed lines and grey shadows in the graphs indicate quantiles (i.e., median and 25^th^, 75^th^ percentiles) of sham-operated animals across all timepoints, statistical comparisons to sham groups were performed for animals of the same timepoint. Detailed time course analyses of sham-operated animals are provided in the Additional file 1: Fig. S1. *p < 0.05, **p < 0.01, ^#^*p* < 0.05, ^##^* p* < 0.01, ^###^* p* < 0.001 vs. sham. *n* = 5 (sham)/timepoint, *n* = 7 (HI)/treatment and timepoint

First, we evaluated the impact of HT on overall microglia activation by immunohistochemistry for Iba-1 (Fig. 3). Iba-1 immunoreactivity did not differ compared to sham animals 1 day after injury (Fig. 3a). However, time course analyses demonstrated that microglia activation peaks 3 days after HI (Fig. 3a–c). In accordance with observed regional differences in HI-induced brain injury (Fig. 2), Iba-1 immunoreactivity was highest in the hippocampus, revealing significant differences between HI-injured and sham control animals 3 and 7 days after HI, which was, however, independent of treatment (Fig. 3b, c). No differences were observed in sham-operated animals between different timepoints and regions (Additional file 1: Fig. S1). To get deeper insight, whether HI-induced neuronal damage and microglia activation are interrelated or two independent events, we performed correlation analyses between the number of TUNEL^+^ cells/%neuronal loss with Iba-1 immunoreactivity (Additional file 1: Fig. S2a). Analyses over both treatment groups and all analyzed brain regions demonstrated a significant positive correlation 3 and 7 days but not 1 day after HI (Additional file 1: Fig. S2a). Separate correlation analyses for NT and HT groups further revealed a more pronounced correlation in HT-treated animals compared to NT 3 days after HI (Additional file 1: Fig. S2b).

**Fig. 4:**
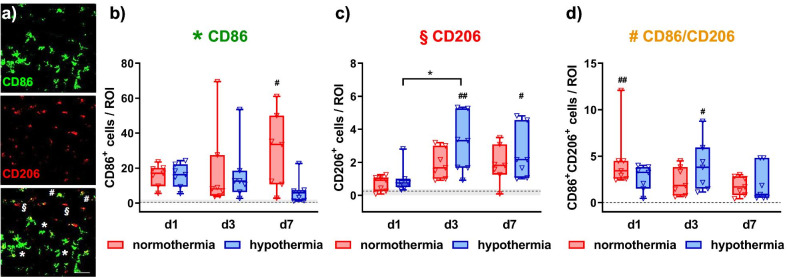
Hypothermia alters temporal expression of typical M1 and M2 marker proteins in the hippocampus. Nine-day-old C57BL/6 mice were exposed to HI followed by 4 h hypothermia or normothermia. Animals were sacrificed and analyzed via immunohistochemistry 1, 3 or 7 days after HI. Myeloid cell polarization was assessed via immunohistochemistry for the prototypical M1 marker CD86 (green) and the M2 marker CD206 (red) in the hippocampus (**a,** representative images are derived from the CA2 region of the hippocampus from an NT-treated animal 3 days after HI. Asterisks, paragraph symbols and rhombi indicate CD86 single, CD206 single and CD86/CD206 double positive cells, respectively; scale bar: 50 µm). CD86 single (**b**), CD206 single (**c**) and CD86/CD206 double (**d**) positive cells were quantified. Dashed lines and grey shadows in the graphs indicate quantiles (i.e., median and 25th, 75th percentiles) of sham-operated animals across all timepoints, statistical comparisons to sham groups were performed for animals of the same timepoint. Detailed time course analyses of sham-operated animals are provided in the Additional file 1: Fig. S4a. *p < 0.05; ^#^* p* < 0.05, ^##^* p* < 0.01 vs. sham. *n* = 5 (sham)/timepoint, *n* = 7 (HI)/treatment and timepoint

To characterize myeloid cells in HI-injured brains more specifically, we assessed expression of two typical marker proteins, associated with classical M1 and alternative M2 activation, i.e., CD86 and CD206, respectively (Fig. 4). As previously described [12], in addition to single positive cells, we also detected a considerable amount of CD86 and CD206 double positive cells (Fig. 4a), even though the number of CD86 single positive cells was highest in all brain regions analyzed (Fig. 4b, Additional file 1: Fig. S3). In the hippocampus, the most severely injured brain region (Fig. 2), we observed an increase of CD86 positive cells between 3 and 7 days after HI in NT-treated animals, resulting in a significant difference compared to sham-operated control mice (Fig. 4b). In contrast, HT-treated animals revealed a decline in the number of CD86^+^ cells, which did not differ compared to healthy sham mice 7 days after injury (Fig. 4b). Furthermore, the amount of CD206 single positive cells rapidly increased between 1 and 3 days after HI in NT and HT animals, with more pronounced effects in HT-treated mice, leading to a significant difference compared to sham control animals 3 and 7 days after HI (Fig. 4c). Elevated numbers of CD86/CD206 double positive cells were detected 1 and 3 days after injury in NT- and HT-treated animals, respectively, while no additional differences were determined between timepoints and treatments (Fig. 4d). The number of CD86 and CD206 single and double positive cells was similar across all timepoints in sham-operated animals (Additional file 1: Fig. S4a).

Of note, the temporal expression of CD86 single positive cells in the hippocampus differed from that in the cortex and thalamus (Additional file 1: Fig. S3). While similar numbers were determined in NT- and HT-treated animals in the hippocampus, HT significantly reduced the number of CD86^+^ cells in the thalamus 1 day after HI (Fig. 4b, Additional file 1: Fig. S3a). Nevertheless, similar to the hippocampus, CD206 single positive cells increased with time in HT-treated animals leading to significantly elevated numbers 7 days after injury in the thalamus (Additional file 1: Fig. S3a). Due to high inter-individual variability in the cortex, no differences were observed for CD86 and CD206 single positive cells in this region (Additional file 1: Fig. S3b). However, similarly as in the hippocampus and thalamus a significantly increased amount of CD86/CD206 double positive cells was detected in NT-treated animals 1 day after injury, which also significantly declined until 7 days after HI comparable to the thalamus (Additional file 1: Fig. S3). Compared to 3 days, a slightly increased number of CD86/CD206 double positive cells was detected in the thalamus of sham-operated animals 1 day after surgery (Additional file 1: Fig. S4b), which was, however, significantly smaller compared to HI-injured animals (Additional file 1: Fig. S3a). No significant time-dependent differences were observed for CD86 and CD206 expression in the cortex of sham animals (Additional file 1: Fig. S4c).

### Hypothermia decreases neonatal HI-induced infiltration of peripheral myeloid cells

**Fig. 5:**
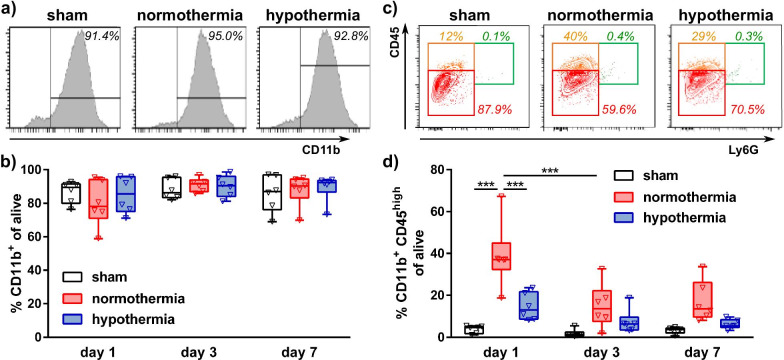
Neonatal HI and HT modulate composition of ex vivo sorted CD11b^ + ^cells. 1, 3 and 7 days after HI or sham operation, CD11b^ + ^cells were isolated via MACS from brains of uninjured sham-operated and HI-injured animals exposed to immediate HT or NT. Flow cytometry with a small aliquot of isolated cells was used to determine purity isolated cells (**a**, **b**) and to characterize the composition of sorted myeloid cells (**c**, **d**). Purity of CD11b^+^ cells was quantified as the percentage of living, i.e., propdidumiodide (PI) negative cells (**a**, **b**). According to Ly6G and CD45 expression, sorted cells were distinguished in resident microglia (red gate: CD11b^+^ CD45 ^int^ Ly6G^−^), peripheral monocytes/macrophages (orange gate: CD11b^+^ CD45^high^ Ly6G^−^) and neutrophils (green gate: CD11b^+^ CD45^high^ Ly6G ^+^) in living (i.e., PI negative) cells (**c, d**). Gates were set according to samples obtained prior to MACS. ***p < 0.001, *n* = 6 animals/timepoint for sham (left and right hemisphere pooled), *n* = 12 animals/timepoint for HI + NT and for HI + HT (2 ipsilateral hemispheres pooled per sample). Histograms and contour plots in **a** and (**b**) are representative for cells isolated 1 day after HI

Since immunohistochemistry is limited to certain brain areas in a small part of the brain, we isolated myeloid cells from the total hemisphere via MACS of CD11b^+^ cells to assess expression of a broad set of genes associated with classical M1 and alternative M2 activation as well as immunomodulatory genes. Cell sorting efficiency was evaluated via flow cytometry, revealing a mean purity of 87.2 ± 8.7%, which did not differ between all groups analyzed (Fig. 5a, b). However, further detailed analyses of the composition of sorted cells according to their CD45 expression, enabling differentiation between brain resident microglia (CD45^int^) and myeloid cells derived from the periphery (CD45^high^), revealed differences between sham and HI-injured animals but also between NT and HT-treatment (Fig. 5c, d). Surprisingly, we hardly detected neutrophils in sorted CD11b^+^ cells. However, comparison of standard isolation procedures for flow cytometry (Additional file 1: Fig. S5a) with cell isolation for MACS according to the manufacturer’s instruction (Additional file 1: Fig. S5b) revealed that the isolation and tissue homogenization procedure for MACS depletes almost all neutrophils (Additional file 1: Fig. S5b,c). While the majority of sorted cells in sham animals were identified as resident microglia, a considerable amount of peripheral infiltrated macrophages was detected in sorted cells of HI-injured animals (Fig. 5c, d, Additional file 1: Fig. S5c). Importantly, the pronounced increase of peripheral macrophages in NT-treated HI-injured animals was significantly reduced by HT 1 day after HI (Fig. 5d). Furthermore, this early increase in macrophages rapidly declined 3 days after injury in both groups (Fig. 5d).

### Hypothermia differentially modulates CD86 and CD206 expression in myeloid cells of the total hemisphere

**Fig. 6:**
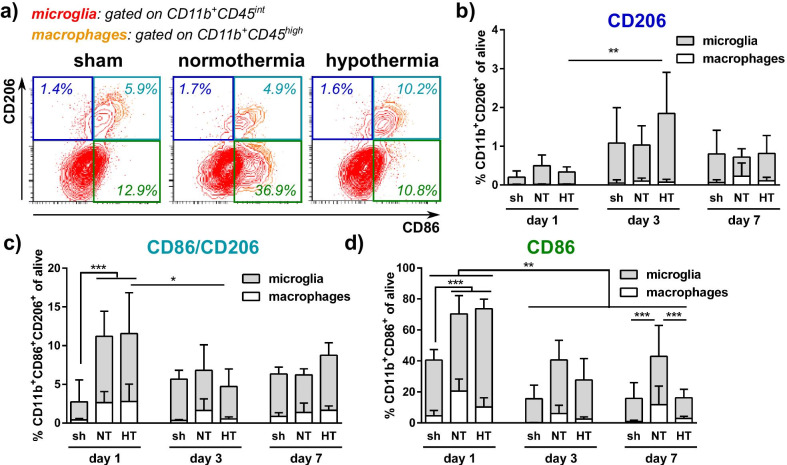
Hypothermia differentially modulates CD86 and CD206 expression in myeloid cells of the total hemisphere. Nine-day-old C57BL/6 mice were exposed to HI followed by 4 h HT or NT. Animals were sacrificed and analyzed via flow cytometry 1, 3 or 7 days after HI. CD11b^+^ cells were isolated via MACS from brains of uninjured sham-operated and HI-injured animals. Flow cytometry with a small aliquot of isolated cells was used to analyze the frequency of CD86^+^ (green gate), CD206^+^ (blue gate) and CD86/CD206^+^ cells (turquoise) cells (**a**, gates were set according to fluorescence minus one controls). Quantification of these subsets (**b**–**d**) was performed in macrophages (CD11b^+^CD45^high^) and resident microglia (CD11b^+^CD45^int^). *p < 0.05, **p < 0.01, ***p < 0.001, *n* = 6 animals/timepoint for sham (left and right hemisphere pooled), *n* = 12 animals/timepoint for HI + NT and for HI + HT (2 ipsilateral hemispheres pooled per sample). Contour plots in **a** are representative for cells isolated 7 days after HI

To correlate our findings derived from region-specific immunohistochemistry for CD86 and CD206 expression, we further quantified the amount of single and double positive cells in ex vivo sorted CD11b^+^ cells differentiating between microglia and peripheral macrophages (Fig. 6a). Similar to results obtained via immunohistochemistry, the amount of CD86 single positive cells was higher compared to CD206 single and double positive cells at all timepoints (Fig. 6). Comparable to observations in the hippocampus, the increase in CD206 single positive cells 3 days after HI was also detected in sorted myeloid cells of the total brain (Fig. 6b). Furthermore, an acute increase of CD86/CD206 double positive cells was determined in HI-injured animals compared to healthy sham controls, which was independent of treatment (Fig. 6c). Confirming observations in the hippocampus at day 1, CD86 single positive cells were increased in HI-injured animals compared to sham controls independent of treatment, while HT-treated animals had reduced numbers of CD86 positive cells 7 days after HI compared to NT-treated mice (Fig. 6d).

In contrast to immunohistochemistry, flow cytometry analyses enabled the differentiation between effects on resident microglia and macrophages derived from the periphery. On the one hand, both cell types partly revealed similar regulations, e.g., a significant increase of CD86/CD206 double positive cells in HI-injured animals 1 day after HI, independent of treatment (Fig. 6c, Additional file 1: Table S2). On the other hand, the acute increase in CD86^+^ cells in the total CD11b^+^ cell population of NT-treated HI-injured mice was mainly based on a significant increase in peripheral macrophages, which was reduced in HT-treated animals (Fig. 6d, Additional file 1: Table S2). Of note, the overall increase in CD206 single positive cells particularly in HT-treated animals was mainly due to an increase of these cells in the microglia population, while these cells were hardly detectable in peripheral macrophages (Fig. 6b, Additional file 1: Table S2).

### Hypothermia decreases HI-induced upregulation of a broad set of inflammatory genes associated with classical M1 and alternative M2 activation

**Fig. 7:**
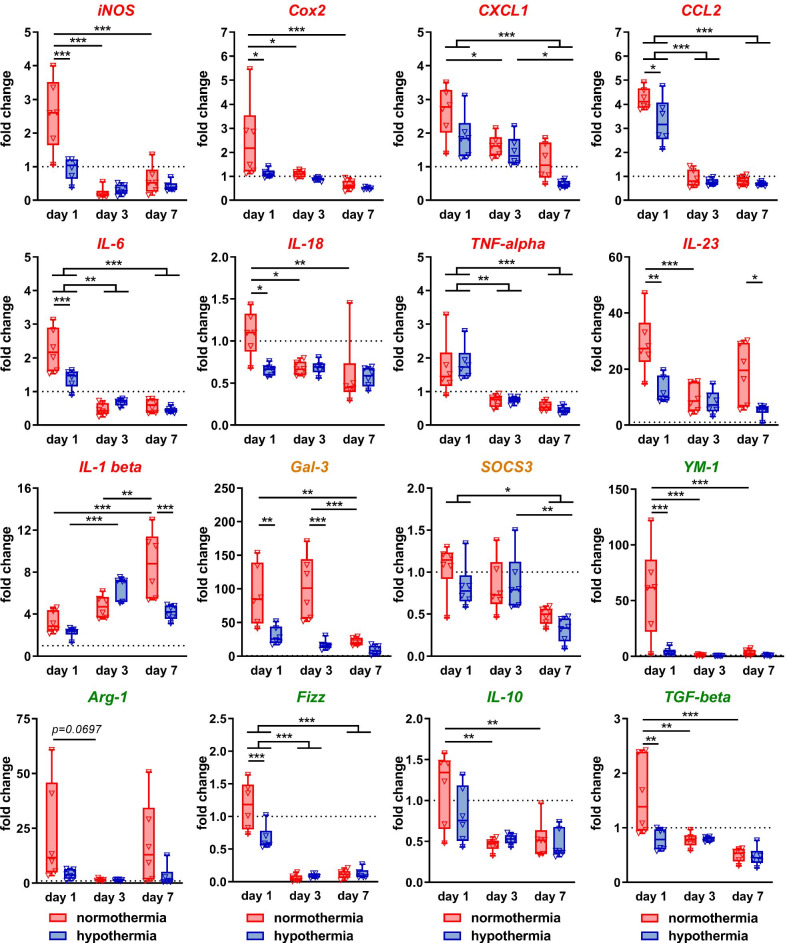
Hypothermia suppresses HI-induced upregulation of a large number of inflammatory genes in myeloid cells. To investigate a large set of genes associated with different myeloid cell polarization states, mRNA expression analyses was performed via real time PCR on MACS-sorted CD11b^+^ cells from brains of C57Bl/6 mice exposed to HI at P9 followed by immediate HT or NT. Uninjured sham-operated animals served as controls. A broad set of typical M1 (red heading), immunoregulatory (orange heading) and M2 (green heading) marker genes were analyzed. Beta-2-microglobulin served as housekeeping gene and fold change values compared to sham-operated control animals were calculated. *p < 0.05, **p < 0.01, ***p < 0.001, *n* = 6 animals/timepoint for sham (left and right hemisphere pooled), *n* = 12 animals/timepoint for HI + NT and for HI + HT (2 ipsilateral hemispheres pooled per sample)

To investigate a larger set of molecules associated with different myeloid cell polarization states, we performed gene expression analyses on MACS-sorted CD11b^+^ cells. Genes were assigned to M1 (*iNOS, Cox2, CXCL-1, CCL-2, IL-6, IL-18, TNF-alpha, IL-23, IL-1beta*), immunomodulatory markers (*Gal-3, SOCS3*) and M2 (*YM-1, Arg-1, Fizz-1, IL-10, TGF-beta*) according to previous reports [10–12, 14, 16, 19]. Interestingly, compared to sham animals, all analyzed genes were immediately upregulated after HI, which was, however, independent of myeloid cell classification (Additional file 1: Table S3). As such, the most upregulated genes compared to uninjured sham-operated animals were the immunomodulatory marker *Gal-3* (mean fold change: 91.6), the M2 marker *YM-1* (mean fold change 58.5) and the M1 marker *IL-23* (fold change: 29.1) (Additional file 1: Table S3, Fig. 7). Furthermore, for the majority of genes we detected a significantly increased expression in NT-treated HI-injured animals compared to HT-treated mice 1 day after HI, independent of myeloid cell classification (i.e., *iNOS, Cox2, CCL-2, IL-6, IL-18, IL-23, Gal-3, YM-1, Fizz-1, TGF-beta,* Fig. 7). This acute and partially strong increase rapidly declined 3 days after HI to levels of sham animals or even below (Fig. 7). However, we also identified a few exceptions, e.g., *IL-1beta* and *IL-23* revealed a delayed and a secondary increase, respectively, in NT-treated animals 7 days after HI, which was significantly reduced by HT (Fig. 7). Furthermore, Gal-3 displayed a sustained increase in NT-treated animals until 3 days after HI, which was significantly decreased by HT and which declined to the level of HT-treated animals 7 days after injury and intervention (Fig. 7).

## Discussion

Therapeutic hypothermia (HT) is the standard therapy for the treatment of neonatal encephalopathy of presumed hypoxia–ischemia (HI). However, 30% of cooled infants still suffer from major neurological problems [3], because of a short therapeutic window (first 6 h of life) and a limited efficacy in more severe cases [4]. In spite of a large body of pre-clinical studies suggesting adjuvant therapies, clinical translation failed until now. Therefore, a better knowledge of hypothermia’s effector mechanisms is needed to guide the rational design of combination treatments. Myeloid cells play a key role in HI pathophysiology, revealing a high phenotypic plasticity either participating in progression or resolution of injury-induced inflammation. In the present study, we demonstrate that HT-induced neuroprotection develops over time, which is preceded by a strong and acute suppression of HI-induced upregulation of a large number of inflammatory genes in myeloid cells, associated with, both, classical M1 and alternative M2 activation. Furthermore, HT selectively inhibited secondary increases of pro-inflammatory cytokines and decreased HI-induced infiltration of peripheral myeloid cells.

Despite of a large number of studies including our own, demonstrating significant neuroprotection at delayed timepoints, i.e., 7–8 days after neonatal HI [5, 9, 23–26], reports on HT effects on early cellular degeneration are still contradicting. On the one hand, reduced apoptosis (i.e., caspase-3 and TUNEL) was observed 6 h after HI in a piglet and in a rat model [27, 28]. On the other hand, several reports could not demonstrate significant protection from acute cell death in severely affected brain regions, i.e., the hippocampus. For instance, HT was significantly neuroprotective in the caudate nucleus but not in the hippocampus in a severe neonatal HI piglet model 48 h after HI [29]. These data are in line with our previous report in neonatal rats, where we also did not detect significant protection by HT from acute cellular degeneration 48 h after HI [30]. Furthermore, no obvious differences were observed for the number of apoptotic cells between NT and HT-treated animals 1 day after HI in 7-day-old rats [31]. In support of this, in the present study we also did not observe significant differences in the number of TUNEL-positive cells 1 day after HI. However, 3 days after HI a slight, though not significant, reduction of cellular degeneration was detected in the hippocampus, which resulted in a significantly decreased neuronal loss 7 days after HI. Together, these data indicate that neuroprotective effects of HT develop over time and become most obvious at the subacute disease phase.

According to previous clinical and pre-clinical reports suggesting that HT is more efficient in moderate than in severe injury cases [26, 32], we used our previously established model in 9-day-old C57BL/6 mice. This model produces moderate to severe hippocampal brain injury, which can be significantly attenuated by the HT protocol, applied in the present study [5, 9]. Therefore, our experimental paradigm is particularly suitable to investigate mechanisms underlying effector mechanisms of HT. In contrast to rats, it is hardly possible to produce more severe injury also in other brain regions (i.e., cortex and thalamus) by, e.g., increasing temperature or hypoxia severity due to an inacceptable high mortality of 50% in C57BL/6 mice in these severe injury models [5, 22]. Though mild injury in the cortex and thalamus may provide first insights into therapeutic efficacy of HT in mild HIE, valid conclusions would need larger sample sizes due to small effect sizes of the HI insult in these regions. Previous preclinical reports in rats, however, already provide first indications that HT may also protect from mild HI-induced brain injury [33], which is also increasingly discussed in the clinical setting [34, 35]. In addition to the severity of the HI insult, the present HT protocol may be important for interpretation of results. While clinical studies and large animal models suggested that 72 h HT provides best therapeutic effects [3, 32, 36], a consensus about the optimal cooling protocol in neonatal rodents, particularly in mice, is still missing. However, the vast majority of rodent studies applied short HT (4–5 h), all of them showing neuroprotection, though with different effect sizes [5, 9, 23, 24, 37, 38]. Considering pronounced differences in physiology between rodents and large animals/humans, longer durations may not necessarily increase neuroprotective effects. This is supported by the few studies, applying longer HT durations in rodents, which did not show an additional benefit of longer cooling [26, 39–41]. Nevertheless, these rodent studies also emphasize the utmost importance of large animal models for translational research, which was not the main purpose of the present study. The major aim was the identification of cellular and molecular mechanisms, underlying neuroprotective effects of HT.

Our time course analyses of inflammatory processes specifically focusing on myeloid cells, i.e., microglia and infiltrated myeloid cells from the periphery, demonstrate a distinct temporal and spatial pattern of cell activation and phenotypic plasticity. In line with limited neuroprotection 3 days after injury, Iba-1 immunoreactivity was not modulated by HT. These data are supported by correlation analyses, which demonstrated a significant positive correlation between apoptosis/neuronal loss and Iba-1 immunoreactivity in HT-treated animals 3 days after HI. Though our experimental paradigm discussed above may provide an explanation for limited effects of HT on Iba-1 reactivity, it should also be taken into account that HT may only delay HI-induced neurodegenerative processes [42] and possibly associated inflammatory responses. Our results of Iba-1 immunoreactivity rather argue against this hypothesis, because, both, NT and HT-treated animals showed the same peak in the first week. Nevertheless, the continuous increase of CD86 and IL-1beta expression until 7 days, observed in NT-treated animals, may indicate ongoing secondary and tertiary inflammatory mechanisms [43], which might be delayed by HT. Therefore, future studies should include later timepoints of analyses. In spite of a strong correlation between neuronal loss and Iba-1 in both treatment groups 7 days after HI, neuroprotective effects of HT were not accompanied by differences in Iba-1 immunoreactivity at this timepoint. These results indicate that other mechanisms also contribute to HT-induced neuroprotection in the sub-acute disease phase. Furthermore, in view of the enormous heterogeneity of this cell population, sole Iba-1 immunoreactivity might not be suitable for conclusions about microglia/myeloid cell phenotype and function. This is supported by our gene expression analyses in sorted CD11b^+^ cells and by several reports demonstrating that myeloid cell populations differ in morphology and inflammatory profile, not only depending on the time course of disease but also on brain regions [44–46].

Regional differences also need to be considered for interpretation of our results derived from flow cytometry and immunohistochemistry for the M1 marker CD86 and the M2 marker CD206. For instance, while a second increase in CD86 single positive cells between 3 and 7 days was specifically detected in the hippocampus, a gradual decrease was observed in the myeloid cell population obtained from the total hemisphere. These findings support that microglia/macrophages responses to the HI insult but also to HT may differ between different brain regions. Nevertheless, both, immunohistochemistry and flow cytometry revealed that HI induces an acute increase in CD86 single and CD86/CD206 double positive myeloid cells. HT significantly reduced the amount of CD86^+^ cells 7 days after HI and increased the number of CD206^+^ cells 3 days after HI. Even though these data indicate that HT modulates myeloid cell polarization from a pro-inflammatory M1 phenotype to an anti-inflammatory M2 phenotype, the detection of double positive cells is in line with previous reports and highlights that this strict M1/M2 classification concept might oversimplify the complexity of myeloid cell responses in vivo [12]*.* Another crucial aspect, which needs to be considered, but was not in the primary scope of the present study, is the impact of sex. Though not powered enough, preliminary exploratory sex-stratified analyses of the present immunohistochemistry results revealed an increased neuroprotective effect of HT in the hippocampus of females (Additional file 1: Fig. S6a–c), supporting previous reports [38]. With regard to inflammatory responses, an overall increased number of CD86^+^ cells was detected in males 3 days after HI (Additional file 1: Fig. S6e), which is in line with previous work in a model of neonatal ischemic stroke, demonstrating an increased pro-inflammatory profile in myeloid cells, isolated from male mice [17]. In support of this, it was recently shown that depletion of myeloid cells provides neuroprotection in males but not in females [47]. Females revealed larger numbers of CD86/CD206 double positive cells, particularly in HT-treated animals (Additional file 1: Fig. S6g). However, clear-cut conclusions about potential sex-differences in myeloid cell responses to HI and HT will require further studies with larger sample sizes to combat the well-known model-associated variability.

While neuroprotective effects of HT became particularly evident at later timepoints, our myeloid cell specific gene expression analyses uncovered early and very distinct molecular regulations in myeloid cells in response to HI and HT. Almost all analyzed genes including typical M1, M2 and immunoregulatory markers were strongly upregulated in NT-treated HI-injured animals 1 day after the insult. Compared to previous studies on total tissue lysates, temporal regulations detected in the present work seem to differ. For example, Hellström-Erkenstam et al. observed the most pronounced upregulation for the majority of genes already 6 h after HI, which rapidly decreased within 24 h, resulting in no differences between contra- and ipsilateral hemispheres for most of the genes [12]. Furthermore, even though our previous mRNA expression analyses in total tissue lysates also showed reductions below sham levels for, e.g., Cox-2 and iNos, other genes (e.g., IL-6 and TNF-alpha) were upregulated 7 days after HI [13], which is in contrast to the present results obtained from sorted myeloid cells. These discrepancies demonstrate the advantage of cell-specific analysis performed in the present study and suggest that other CNS cell types also contribute to the inflammatory response following neonatal HI. Nevertheless, the elegant possibility of selective analysis of ex vivo sorted myeloid cells is limited to draw conclusions, whether regulations were caused by peripheral monocytes/macrophages or resident microglia. Indeed, our cell-type-specific flow cytometry analyses for CD86 and CD206 revealed that the acute increase in CD86^+^ cells in NT treated HI-injured mice in the total CD11b cell population was mainly based on a significantly increased expression in peripheral macrophages. Furthermore, the overall increase of CD206 single positive cells, particularly in HT-treated animals, was mainly due to an increase of these cells in the microglia population, while these cells were hardly detectable in peripheral macrophages. Differential immune cell composition and different effects on different cell populations most likely also explain the observed decline below sham levels for several genes 3 and 7 days after HI. Finally, the observed reduced infiltration of peripheral macrophages in HT-treated animals 1 day after HI, may partly explain HT-induced downregulation of the majority of inflammatory genes at this early timepoint.

In addition to the acute effects of HT on a broad variety of inflammatory genes, our gene expression screening uncovered potentially important effector molecules mediating HT-induced neuroprotection on delayed neuronal death after neonatal HI. The late increase in IL-1beta and IL-23 indicates that HI induces selective long-lasting inflammatory alterations in myeloid cells, which is supported by previous studies demonstrating a gradual and sustained increase of IL-1beta from 1 to 14 days after HI [48, 49]. Furthermore, the secondary increase in IL-23 seems to be an important mechanism considering the pro-inflammatory role of this cytokine in several inflammatory diseases [50] and cerebral ischemia, where IL-23p19 knockdown prevented delayed cerebral ischemic injury by dampening ischemia-induced inflammation [51]. Considering that HT suppressed these delayed inflammatory responses, our present findings suggest that myeloid cell derived IL-1beta and IL-23 play an important role in subacute evolvement of HI-induced brain injury, which can be attenuated by acute HT.

In view of our own and other studies, either demonstrating interaction or non-additive effects of adjuvant therapies [6–9], the present study results provide potential mechanistic insights. We have previously shown that mesenchymal stem cells (MSC) given 3 days after HI and HT are less efficient compared to MSC given to NT-treated mice. As a potential mechanism we identified modulation of MSCs with an increased pro-inflammatory and reduced regenerative profile due to the altered brain environment after HT. Interestingly, while we did not detect any differences between NT and HT in myeloid cells for the majority of genes 3 days after, Gal-3 was the only molecule, revealing a sustained and robust expression (fold change > 100) 3 days after HI, which was strongly downregulated by HT. The role of the immunomodulatory molecule Gal-3 in ischemic brain injury is still controversially discussed, either providing neuroprotection in adult brain injury or promoting neurodegeneration in neonatal HI-induced brain injury [52, 53]. This is further complicated by the fact that Gal-3 is expressed in resident microglia but also peripheral monocytes/macrophages. Nevertheless, Gal-3 is supposed to be important for immunomodulatory functions of MSCs [54, 55]. Considering our previous results of MSC-modulation by the brain environment 3 days after HI and HT, a reasonable hypothesis for future studies might be that myeloid cell-secreted Gal-3 is needed for immunosuppressive function of MSCs and/or to enhance MSCs’ own Gal-3 expression in vivo. Therefore, either later timepoints of stem cell therapy, i.e., 7 days after HI, when Gal-3 expression is at similar levels in both, NT and HT conditions, or additional therapies to promote Gal-3 expression after HT might present new approaches to improve outcome of stem cell therapy in the obligate clinical setting of HT.

## Conclusions

In the present study, we demonstrate that HT-induced neuroprotection develops over time. Neuroprotective effects are preceded by a strong and acute suppression of HI-induced upregulation of a large number of inflammatory genes in myeloid cells, associated with both, classical M1 and alternative M2 activation. These findings support the overall criticism on the previously suggested strict M1/M2 classification based on single molecule expression. In addition, we demonstrate that HT significantly decreases HI-induced infiltration of peripheral myeloid cells and suppresses the secondary increase of specific pro-inflammatory genes. Our study highlights the importance of time course analyses and the need to consider the complex interplay between peripheral and CNS-resident immune cells. Future studies should perform selective analyses on specific cell types differentiating between resident microglia and different peripheral leukocyte subsets to identify specific targets for therapeutic intervention. Nevertheless, the pronounced early and delayed immunomodulatory effects represent important short- and long-term effector mechanisms of HT, which most likely combine to promote neuroprotection. We identified selective target molecules and potential new therapeutic windows that may pave the way for the rational design of adjuvant therapies.

## Supplementary Information

**Additional file 1.** Additional figures and tables.

## Data Availability

All data generated or analyzed during this study are included in this published article and its supplementary information files.

## Abbreviations

Arg1: Arginase 1
CCL2: Chemokine (C–C motif) ligand 2
Cox2: Cyclooxygenase-2
CXCL1: Chemokine (C–X–C motif) ligand 1
Fizz1: Resistin-like alpha
Gal-3: Lectin, galactose binding, soluble 3
HI: Hypoxia–ischemia
HT: Hypothermia
IL-10: Interleukin-10
IL-18: Interleukin-18
IL-1 beta: Interleukin-1 beta
IL-23: Interleukin 23, alpha subunit p19
IL-6: Interleukin-6
iNos: Nitric oxide synthase 2
NT: normothermia
MACS: Magnetic activated cell sorting
SOCS3: Suppressor of cytokine signaling 3
TGF beta: Transforming growth factor beta-1
TNF alpha: Tumor necrosis factor alpha
YM1: Chitinase-like protein 3
