# Supplementary material for: Hypothermia modulates myeloid cell polarization in neonatal hypoxic–ischemic brain injury
Source: J Neuroinflammation. 2021 Nov 13;18:266. doi: 10.1186/s12974-021-02314-9 (PMC8590301; doi:10.1186/s12974-021-02314-9)
Supplement: Supplementary file 1 — Additional file 1. Additional figures and tables. [file 12974_2021_2314_MOESM1_ESM.pdf]

Additional file 1

Hypothermia modulates myeloid cell polarization in neonatal hypoxic-ischemic brain injury

Marina Seitz[1,2], Christian Köster[1,2], Mark Dzietko[1,2], Hemmen Sabir[3,4], Meray Serdar[1,2], Ursula Felderhoff-Müser[1,2], Ivo Bendix[1,2#], Josephine Herz[1,2*#]*

[1] Department of Pediatrics I, Neonatology & Experimental Perinatal Neurosciences, University Hospital Essen, University Duisburg-Essen, Essen, Germany

[2] Center for Translational Neuro-and Behavioral Sciences (C-TNBS), University Hospital Essen, University Duisburg-Essen, Essen, Germany

[3] Department of Neonatology and Pediatric Intensive Care, Children's Hospital, University of Bonn, Bonn, Germany

[4] German Centre for Neurodegenerative Diseases, Bonn, Germany

**equal contribution*

[#]Corresponding authors:

Josephine Herz: josephine.herz@uk-essen.de

Ivo Bendix: ivo.bendix@uk-essen.de

Figures: S1-S6

Tables: S1-S3

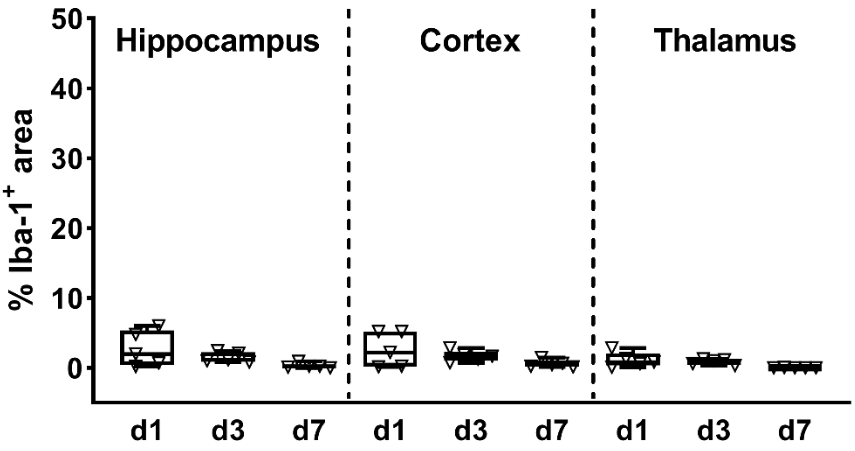

Fig. S1: Iba-1 immunoreactivity does not change over time in sham-operated animals. Nine-day-old C57Bl/6 mice were exposed to sham operation, i.e. unilateral ligation of the common carotid artery followed by analyses of microglia activation via immunohistochemistry 1, 3 and 7 days after HI in the depicted brain regions. n=5/time point. Quantiles of pooled data from all time points are presented as dashed lines (median) and grey shadows (25th and 75th percentiles) in Fig. 3 of the main manuscript.

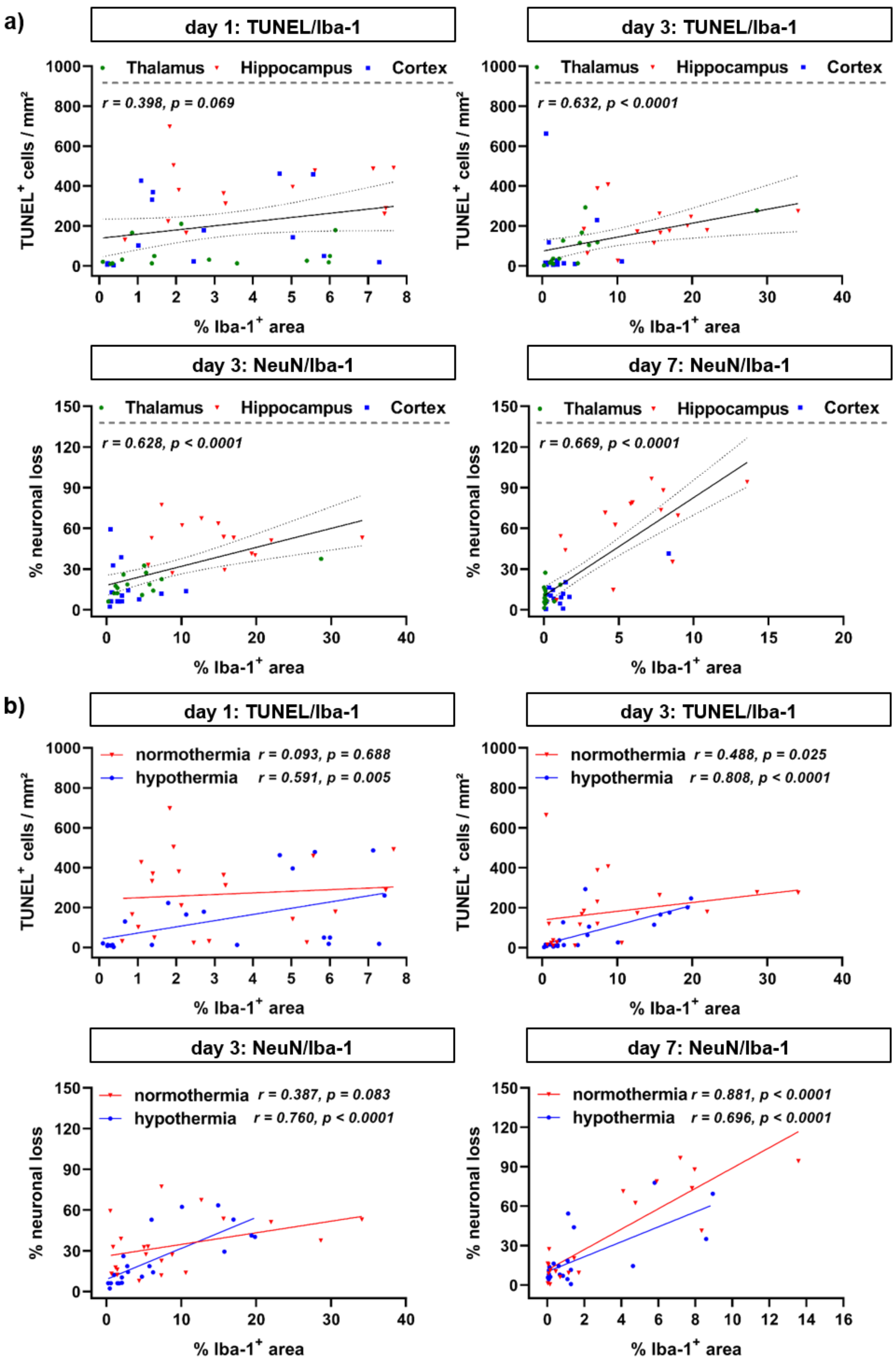

Fig. S2: HI-induced neuronal injury correlates with Iba-1 immunoreactivity 3 and 7 days after HI. Nine-day-old C57BL/6 mice were exposed to HI followed by 4 h hypothermia or normothermia. Animals were sacrificed and analysed via immunohistochemistry 1, 3 and 7 days after HI. Apoptotic cell death, neuronal loss and microglia activation were assessed via immunohistochemistry by TUNEL-labelling, staining of NeuN and Iba-1, respectively.

Correlation analyses was performed on pooled data from both treatment groups and all analysed brain regions, demonstrating a significant and positive correlation 3 and 7 days after injury (**a**). Separate correlation analyses for each treatment group including all analysed brain regions (**b**) revealed a more pronounced correlation in HT-treated animals 3 days after HI. n=7/time point and treatment and brain region. r=correlation coefficient, p=p-value

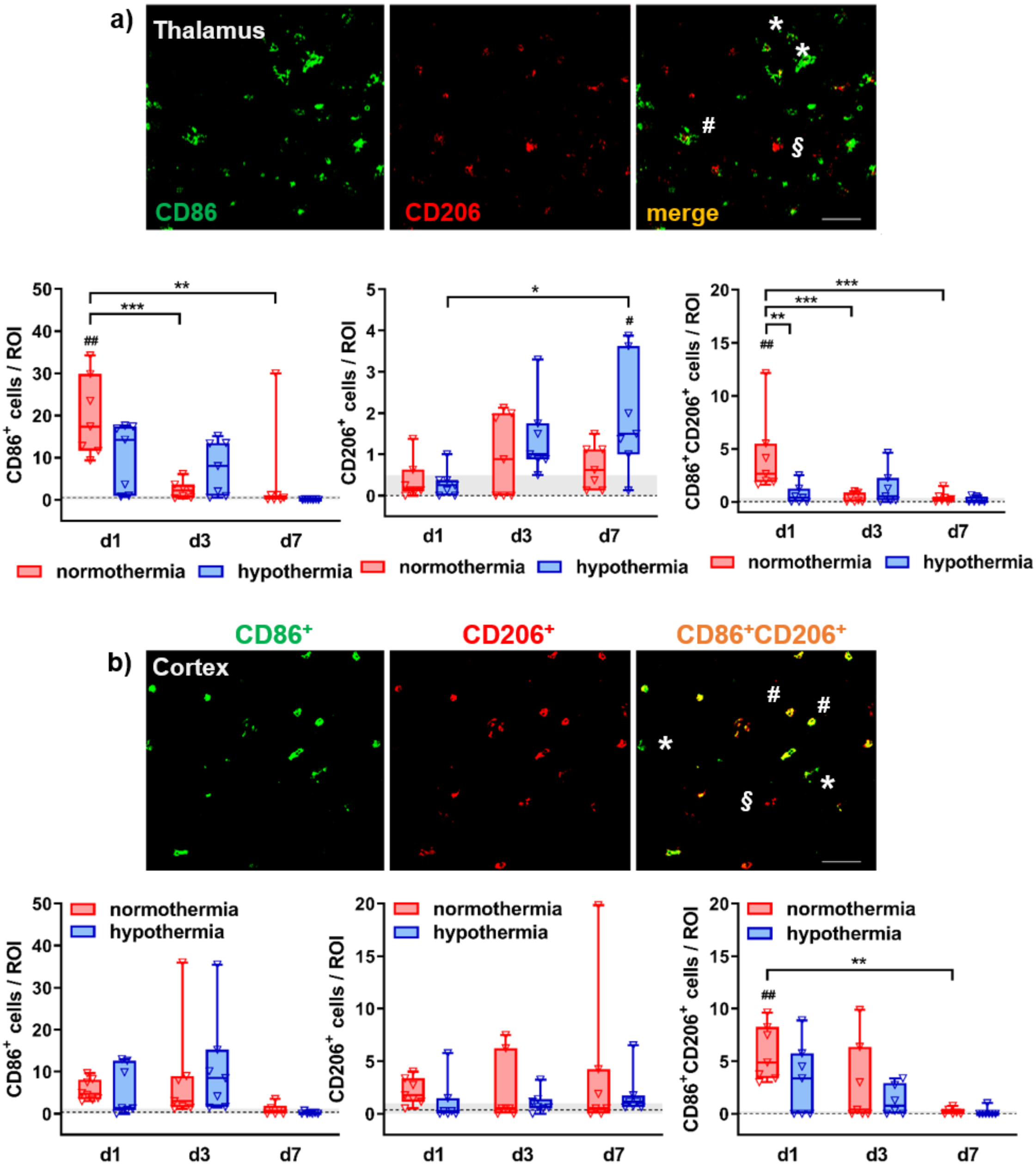

Fig. S3. Impact of HI and hypothermia on expression of typical marker proteins associated with M1 and M2 polarization. Nine-day-old C57BL/6 mice were exposed to HI followed by 4 h hypothermia or normothermia. Animals were sacrificed and analysed 1, 3 or 7 days after HI. Myeloid cell polarization was assessed via immunohistochemistry for the prototypical M1 marker CD86 (green) and the M2 marker CD206 (red) in the cortex (**a**) and thalamus (**b**). CD86 single (left), CD206 single (middle) and CD86/CD206 double (right) positive cells were quantified. Dashed lines and grey shadows in the graphs indicate quantiles (i.e. median and 25th, 75th percentiles) of sham-operated animals across all time points, statistical comparisons to sham groups were performed for animals of the same time point. Detailed time course analyses of sham-operated animals are provided in this Additional file 1:

Fig. S4b,c. *p<0.05; *p<0.05, **p<0.01, ***p<0.001, $^{\#}$p<0.05, $^{\#\#}$p<0.01 *vs*. sham. n=5 (sham)/time point, n=7 (HI)/treatment and time point

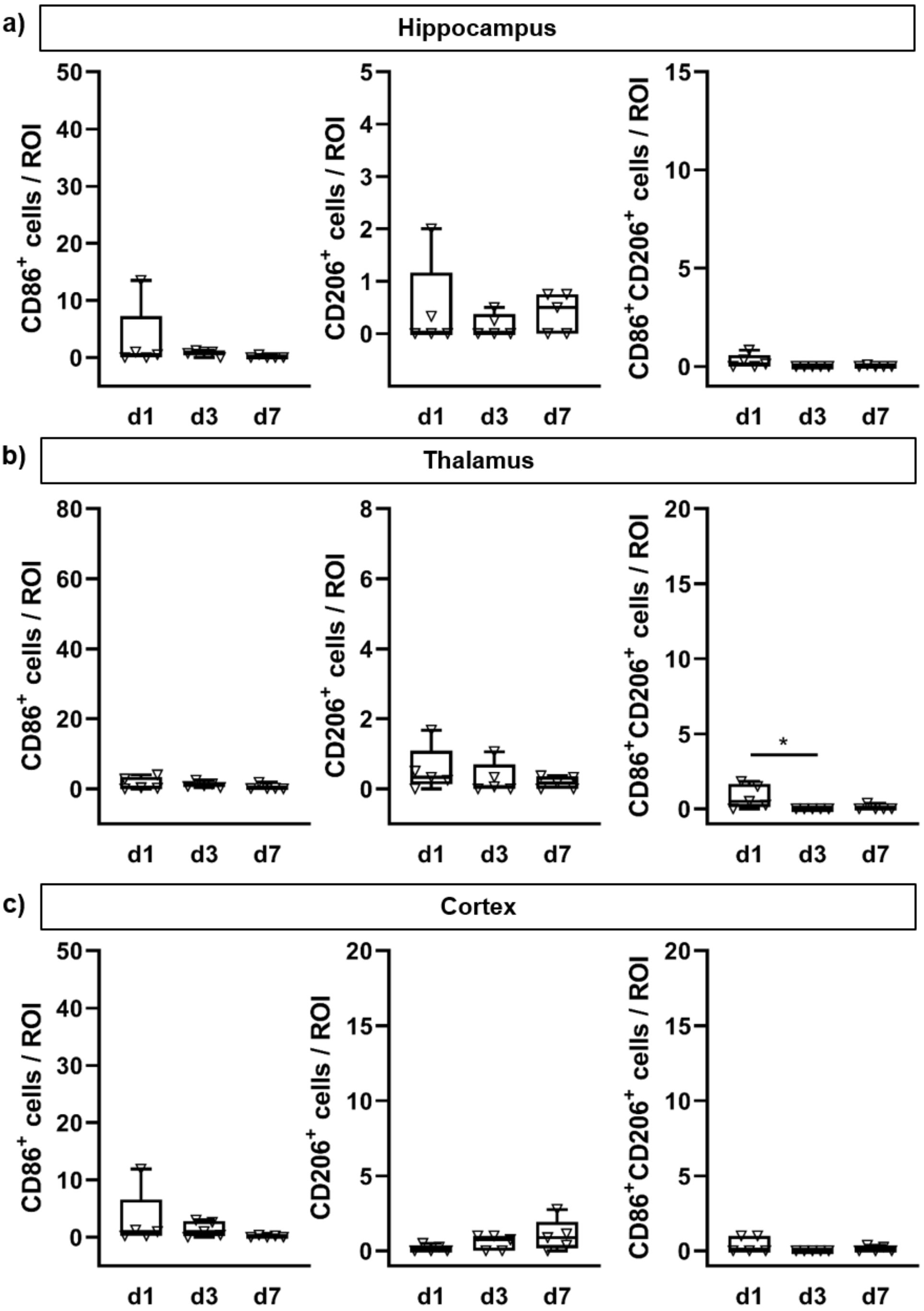

Fig. S4: Microglia polarization is comparable between different time points of analyses in sham-operated mice. Nine-day-old C57Bl/6 mice were exposed to sham operation, i.e. unilateral ligation of the common carotid artery followed by analyses of microglia polarization via immunohistochemistry for the M1 marker CD86 and the M2 marker CD206 1, 3 and 7 days after HI in the hippocampus (**a**), thalamus (**b**) and cortex (**c**). Except of a slightly increased number of CD86/CD206 double positive cells in the thalamus day 1 after surgery (**b**), no significant time-dependent differences were observed for CD86 and CD206 expression in the hippocampus (**a**) and cortex (**c**) of sham animals. n=5/time point. Quantiles of pooled data from all time points are presented as dashed lines (median) and grey shadows (25th and 75th percentiles) in Fig. 4 of the main manuscript and Fig. S3 of this Additional file 1. *p<0.05

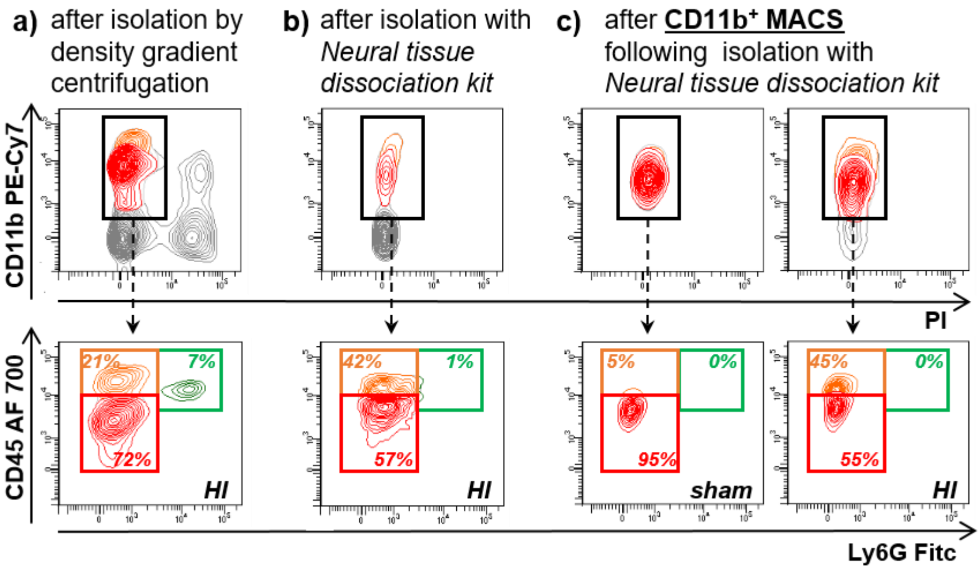

Fig. S5: Immune cell composition after different tissue homogenization and cell isolation procedures. One day after HI, animals were deeply anesthetized by i.p. injections of an overdose chloralhydrate followed by transcardial perfusion with ice-cold PBS and removal of brains. For comparison of the impact of different tissue homogenization and cell isolation procedures on the composition of isolated cells, brain hemispheres were dissected and homogenized through a 70 µm cell strainer (BD Biosciences) by continuous rinsing with 15 mL of cold HEPES-buffered RPMI1640. Brain samples were centrifuged at 400*xg* for 10 min at 18°C. The supernatants were discarded and the pellets were resuspended in 15 mL of 37% Percoll in 0.01 N HCl/PBS and centrifuged at 2800*xg* for 20 min. Myelin was removed and the remaining cell pellet was washed twice in PBS followed by flow cytometry (**a**). For preparation of cell suspensions to be used for magnetic activated cell sorting (MACS) of CD11b$^+$ cells, brain tissues were mechanically and enzymatically dissociated using the Neural tissue dissociation kit followed by myelin removal according the manufacturer's instructions (Miltenyi Biotech, Germany) (**b**). The cell suspension was incubated with anti-CD11b coupled magnetic microbeads followed by magnetic separation of CD11b$^+$ cells. A small aliquot of sorted cells (1-2 x 10^5) was used for flow cytometry to determine the cellular composition via flow cytometry (**c**). Contour plots are representative for at least 3 animals per condition. Dead cells were excluded by staining with propidiumiodide (PI) prior to measurement.

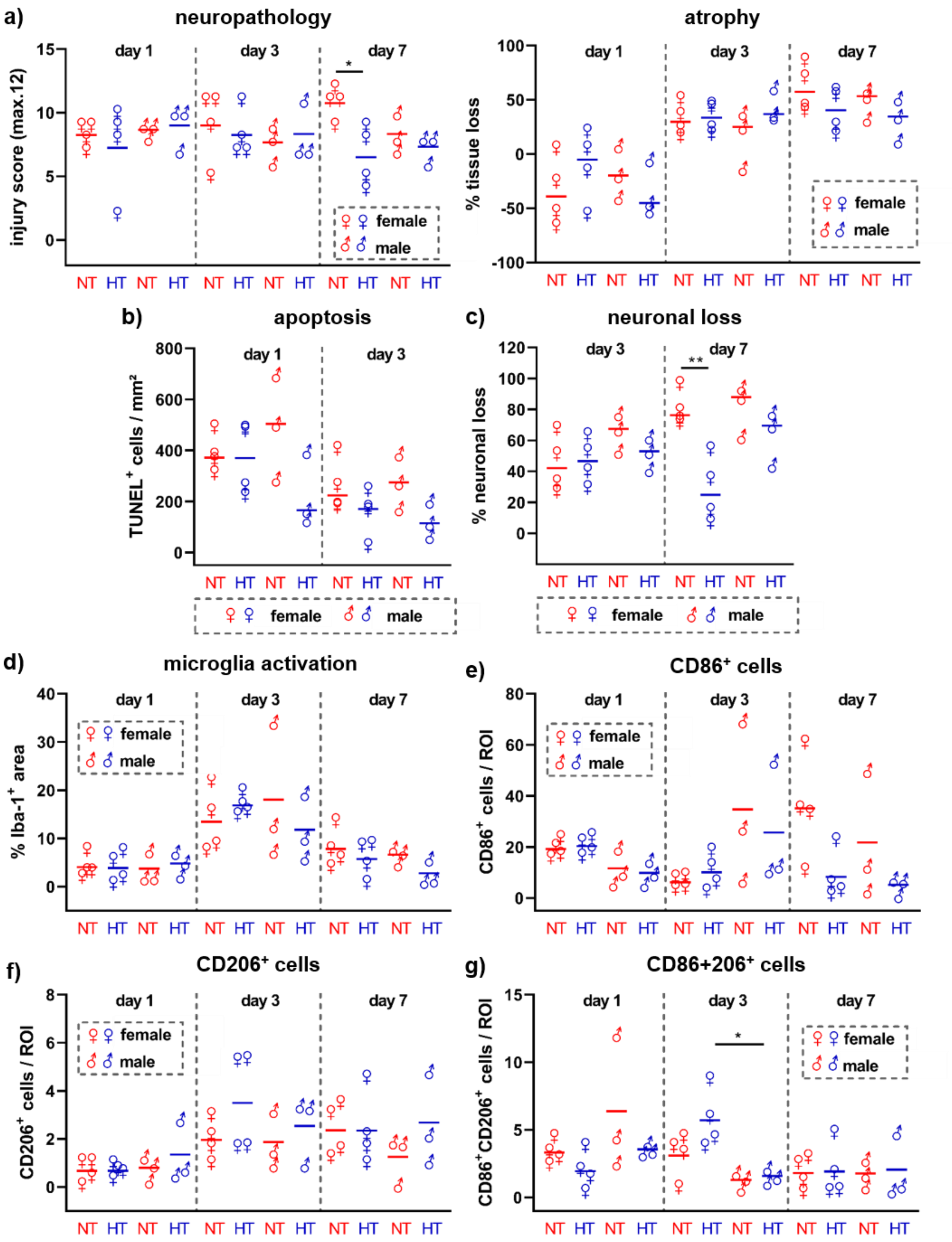

Fig. S6: Sex-stratified analyses of HI-induced hippocampal brain injury and inflammation. Nine-day-old C57BL/6 mice were exposed to HI followed by 4 h hypothermia or normothermia. Brain injury was assessed in cresyl violet stained tissue sections for neurophatological changes and tissue atrophy (**a**). Apoptotic cell death (**b**) and neuronal loss (**c**) were determined via immunohistochemistry by TUNEL labelling (**b**) and staining for NeuN (**c**), respectively. Microglia activation was analysed via immunohistochemistry for Iba-1 (**d**). Myeloid cell polarization was evaluated by quantification of CD86 single (**e**), CD206 single (**f**) and CD86/CD206 double positive (**g**) cells via immunohistochemistry. *p<0.05, **p<0.01, n=3-4/group and time point.

Table S1: TaqMan Assays used for mRNA expression analyses

Gene	Abbreviation	Assay ID
cyclooxygenase-2	Cox2	Mm03294838_g1
interleukin-1beta	IL-1β	Mm00434228_m1
interleukin-6	IL-6	Mm00446190_m1
chitinase-like protein 3	YM1	Mm00657889_mH
nitric oxide synthase 2	iNos	Mm00440502_m1
arginase	Arg1	Mm00475988_m1
transforming growth factor beta-1	TGFβ	Mm01178820_m1
interleukin-18	IL-18	Mm00434226_m1
interleukin-10	IL-10	Mm01288386_m1
tumor necrosis factor alpha	TNFα	Mm00443258_m1
beta-2-microglobulin	B-2m	Mm00437762
chemokine (C-X-C motif) ligand 1	CXCL-1	Mm04207460_m1
chemokine (C-C motif) ligand 2	CCL-2	Mm00441242_m1
interleukin 23, alpha subunit p19	IL-23	Mm00518984_m1
lectin, galactose binding, soluble 3	Gal-3	Mm00802901_m1
suppressor of cytokine signaling 3	SOCS3	Mm01249143_g1
resistin like alpha	Fizz-1	Mm00445109_m1

Table S2: p-Values from cell-type-specific analysis of CD86 and CD206 cell numbers in sorted $CD11b^+$ cells

	microglia			macrophages		
	$CD86^+$	$CD86^+$ $CD206^+$	$CD206^+$	$CD86^+$	$CD86^+$ $CD206^+$	$CD206^+$
Sh d1 vs. NT d1	*0,4902*	**0,008**	*0,9927*	***0,0004***	**0,0308**	*> 0,9999*
Sh d1 vs. HT d1	**0,0045**	**0,0056**	*> 0,9999*	*0,7017*	**0,0175**	*> 0,9999*
NT d1 vs. HT d1	*0,5273*	*> 0,9999*	*0,9999*	*0,0656*	*> 0,9999*	*> 0,9999*
Sh d3 vs. NT d3	*0,1198*	*> 0,9999*	*> 0,9999*	*0,6823*	*0,5681*	*0,9986*
Sh d3 vs. HT d3	*0,8628*	*0,9982*	*0,3955*	*0,9983*	*> 0,9999*	*> 0,9999*
NT d3 vs. HT d3	*0,8864*	*0,9993*	*0,2346*	*0,9737*	*0,76*	*> 0,9999*
Sh d7vs. NT d7	*0,2798*	*> 0,9999*	*0,9977*	***0,0398***	*0,9968*	*0,4227*
Sh d7 vs. HT d7	*> 0,9999*	*0,9786*	*> 0,9999*	*0,9996*	*0,9552*	*0,9995*
NT d7 vs. HT d7	*0,1911*	*0,8753*	*0,999*	*0,1562*	*> 0,9999*	*0,805*
Sh d1 vs. Sh d3	*0,0756*	*0,6263*	*0,2337*	*0,9069*	*> 0,9999*	*0,9998*
Sh d1 vs. Sh d7	*0,0614*	*0,5661*	*0,771*	*0,9663*	*0,9984*	*0,998*
Sh d3 vs. Sh d7	*> 0,9999*	*> 0,9999*	*0,9916*	*> 0,9999*	*0,9962*	*> 0,9999*
NT d1 vs. NT d3	*0,366*	*0,4597*	*0,9056*	***0,0016***	*0,8175*	*0,9683*
NT d1 vs. NT d7	*0,1408*	*0,3353*	*> 0,9999*	*0,1779*	*0,5829*	*0,1361*
NT d3 vs. NT d7	*0,9998*	*> 0,9999*	*0,9121*	*0,696*	*> 0,9999*	*0,7386*
HT d1 vs. HT d3	**< 0,0001**	*0,1174*	**0,0018**	*0,3027*	*0,0307*	*0,9979*
HT d1 vs. HT d7	**< 0,0001**	*0,9804*	*0,9549*	*0,357*	*0,7063*	*0,9479*
HT d3 vs. HT d7	*0,706*	*0,6427*	**0,0526**	*> 0,9999*	*0,751*	*> 0,9999*

Statistical analyses were performed by two-way ANOVA followed by Tukey's posthoc test comparing all groups regardless factors, Sh=sham, NT=HI+normothermia, HT=HI+hypothermia

Table S3: Mean fold change values for mRNA expression of typical M1, M2 and immunoregulatory markers in *ex vivo* sorted CD11b⁺ cells

		day 1 after HI		day 3 after HI		day 7 after HI	
		normo-thermia	hypo-thermia	normo-thermia	hypo-thermia	normo-thermia	hypo-thermai
reg	Gal-3	91,65	30,52	102,96	16,82	21,55	9,07
M2	YM-1	58,55	3,86	1,04	0,49	3,39	0,84
M1	IL-23	29,10	12,49	9,65	8,10	18,50	5,30
M2	Arg-1	22,31	3,97	1,49	1,39	18,08	3,04
M1	CCL-2	4,23	3,29	0,91	0,76	0,81	0,68
M1	IL-1b	3,22	2,29	4,70	6,54	8,72	4,16
M1	CXCL-1	2,65	1,90	1,63	1,45	1,14	0,48
M1	iNOS	2,58	1,05	0,21	0,28	0,60	0,41
M1	Cox-2	2,51	1,12	1,09	0,88	0,64	0,49
M1	IL-6	2,24	1,37	0,47	0,70	0,58	0,46
M1	TNF-a	1,69	1,84	0,72	0,75	0,56	0,45
M2	TGF-b	1,57	0,79	0,78	0,79	0,50	0,47
M2	IL-10	1,15	0,82	0,46	0,53	0,53	0,47
M2	Fizz	1,17	0,66	0,05	0,09	0,11	0,12
reg	SOCS-3	1,05	0,83	0,83	0,88	0,48	0,31
M1	IL-18	1,09	0,66	0,68	0,69	0,60	0,57

0 - 0.5	0.5 - 1.0	1.0 - 2.0	2.0 - 5.0	5.0 - 10.0	10.0 - 20.0	20.0 - 50.0	> 50

Fold change values were calculated relative to sham-operated animals, reg=immunoregulatory markers
